# CDKD: a clinical database of kidney diseases

**DOI:** 10.1186/1471-2369-13-23

**Published:** 2012-04-27

**Authors:** Sanjay Kr Singh, Adeel Malik, Ahmad Firoz, Vivekanand Jha

**Affiliations:** 1 Biomedical Informatics Centre, Post Graduate Institute of Medical Education and Research (PGIMER), Chandigarh, 160012, India; 2 Department of Nephrology, Post Graduate Institute of Medical Education and Research (PGIMER), Chandigarh, 160012, India; 3 School of Computational Sciences, Korea Institute for Advanced Study, Seoul, 130-722, South Korea; 4 School of Chemistry & Biochemistry, Thapar University, Patiala, Punjab, India

## Abstract

**Background:**

The main function of the kidneys is to remove waste products and excess water from the blood. Loss of kidney function leads to various health issues, such as anemia, high blood pressure, bone disease, disorders of cholesterol. The main objective of this database system is to store the personal and laboratory investigatory details of patients with kidney disease. The emphasis is on experimental results relevant to quantitative renal physiology, with a particular focus on data relevant for evaluation of parameters in statistical models of renal function.

**Description:**

Clinical database of kidney diseases (CDKD) has been developed with patient confidentiality and data security as a top priority. It can make comparative analysis of one or more parameters of patient’s record and includes the information of about whole range of data including demographics, medical history, laboratory test results, vital signs, personal statistics like age and weight.

**Conclusions:**

The goal of this database is to make kidney-related physiological data easily available to the scientific community and to maintain & retain patient’s record. As a Web based application it permits physician to see, edit and annotate a patient record from anywhere and anytime while maintaining the confidentiality of the personal record. It also allows statistical analysis of all data.

## Background

Kidney disease is a disorder in which the normal functioning of the kidneys with respect to filtration, reabsorption, secretion, etc., is affected. Chronic kidney disease (CKD) has become one of the most important, chronic, noncommunicable disease epidemics in the world, including India [1].

**Table 1:** General statistics of CDKD records

**Factors**	**Male (%)**	**Female (%)**	**Total (%)**
	**n = 6776**	**n = 3425**	**n =10201**
**Age (mean** ± **SE)**	49.69 ± 16.83	47.20 ± 15.88	48.86 ± 16.56
**Sex (population)**	66.42	33.58	100%
**Age group**			
01–14	206 (3.04)	83 (2.42)	289 (2.83)
15–29	722 (10.66)	410 (11.97)	1132 (11.10)
30–44	1296 (19.13)	879 (25.66)	2175 (21.32)
45–59	2428 (35.83)	1205 (35.18)	3633 (35.61)
≥ 60	2124 (31.35)	848 (24.76)	2972 (29.13)
**Hypertension (**P %, S%**)**	22.00, 39.85	21.63, 36.99	21.88, 38.89
**Diabetes (**%**)**	41.88	32.47	38.72
**BMI (**mean ± SE**)**, kg/m^2^	22.47 ± 9.24	23.29 ± 5.85	22.74 ± 8.27
**Smoking (**%**)**	33.85	7.97	25.16
**Creatnine (**mean ± SE**)**, mg/dL	4.34 ± 2.79	3.90 ± 2.66	4.19 ± 2.76
**eGFR (**mean ± SE**)**, mg/dL	31.90 ± 58.84	28.08 ± 51.57	30.62 ± 56.53

*n* Number of patients, *P* primary, *S* secondary, *SE* standard deviation.

**Table 2:** Comparative diseases statistics of CDKD records

	**Diabetic nephropathy**	**Chronic glomerulonephritis**	**Congenital disease**	**Cystic disease**	**Heredofamilial**	**Obstructive uropathy**
**No. of patients** (%)	3171 (31.09)	1640 (176.85)	96 (0.94)	225 (2.21)	42 (0.41)	433 (4.24)
**Age** (mean ± SE)	57.43 ± 10.54	37.45 ± 16.24	27.34 ± 18.81	46.82 ± 12.96	44.88 ± 14.83	47.10 ± 18.10
**Diabetes type 1,** %	3.25	0.12	0.00	0.89	0.00	1.15
**Diabetes type 2,** %	80.13	3.29	2.08	7.11	4.76	7.39
**Serum creatinine** (mean ± SD)	4.14 ± 2.54	3.87 ± 2.94	3.23 ± 2.67	3.85 ± 2.73	4.36 ± 3.16	4.45 ± 2.69
**Mean of eGFR**	24.34	45.48	55.83	31.64	41.96	26.04
**CKD stage 3–5** (%)	93.95	75.49	63.54	84.44	76.19	89.61
**CKD stage 1–2** (%)	5.74	23.90	35.42	15.56	23.81	9.47
**Diabetes family history** (%)	37.81	8.54	15.63	7.14	7.14	6.00
**Hypertension** (P %, S%)	22.83, 46.70	5.18, 46.65	7.29, 21.88	6.22, 55.11	4.76, 45.24	11.78, 18.94
**Smoking** (%)	30.37	17.74	10.42	19.56	38.10	25.87

**Figure 1:**
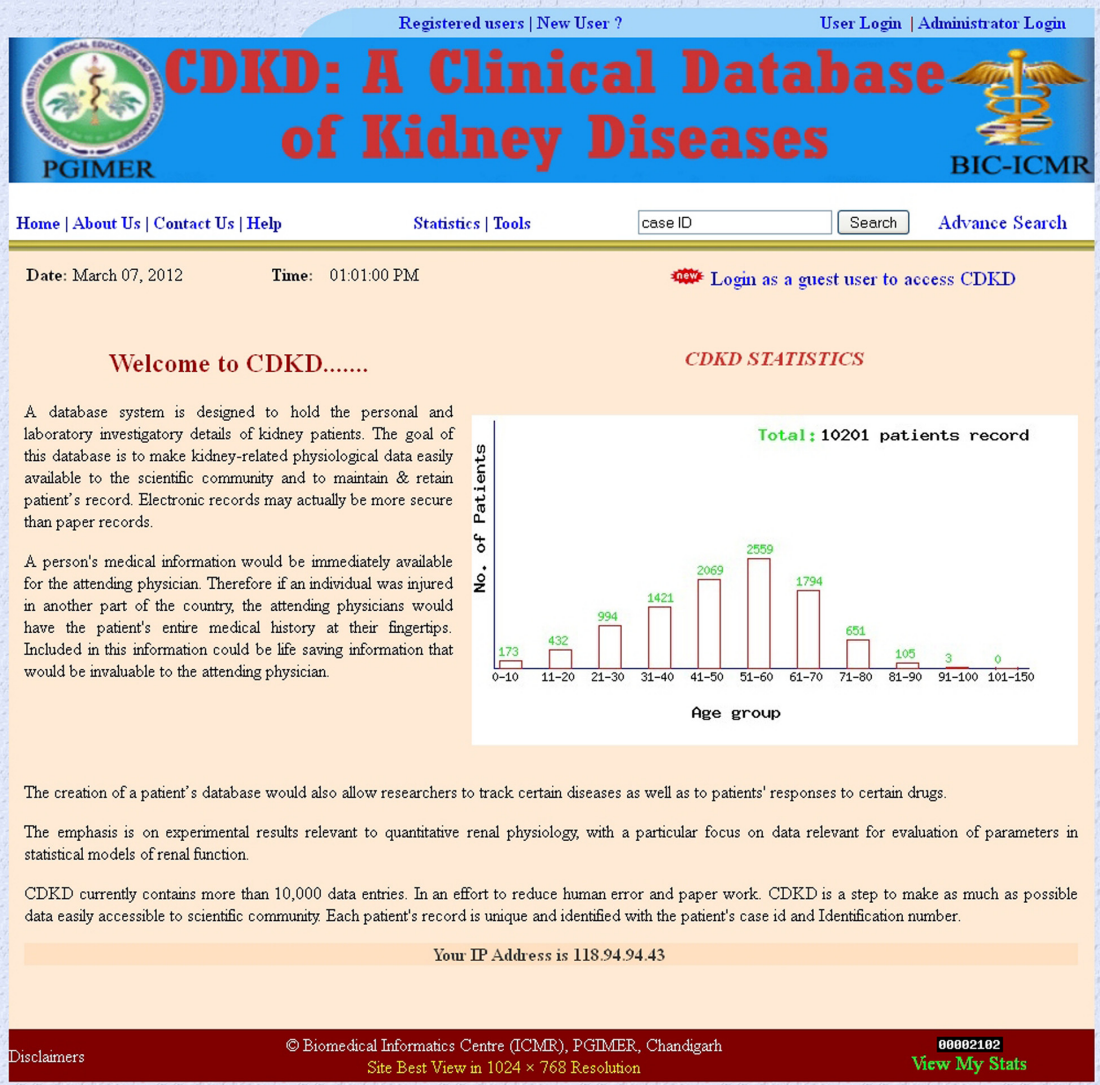
**Home page of CDKD.** Brief description of CDKD is available on this page and a bar chart is also generated dynamically displaying total number of patients (in an age wise manner). In bar chart the X-axis displays number of patients and the Y-axis shows age group. Over the bar, number of patients relevant to age group is displayed in green color.

**Figure 2:**
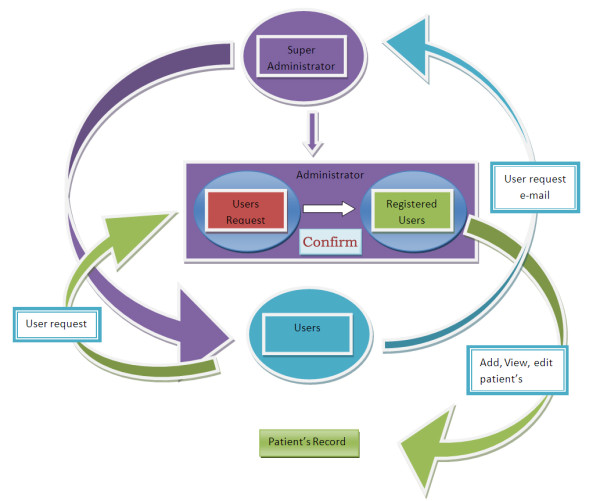
**Showing architecture of patient’s record.** This figure displays patient’s record management system. Patient’s record is divided into three sections; personal details, case details and record history (follow-ups).

**Figure 3:**
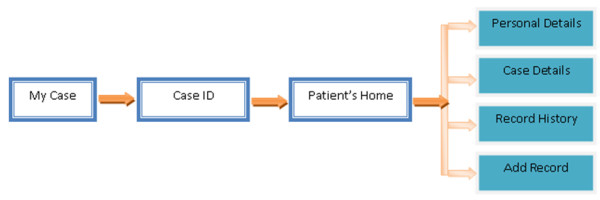
**A flow chart explaining the registration system of CDKD.** This figure explains the registration system of CDKD. This registration system is designed in such a manner so that unauthorized users cannot be registered.

**Figure 4:**
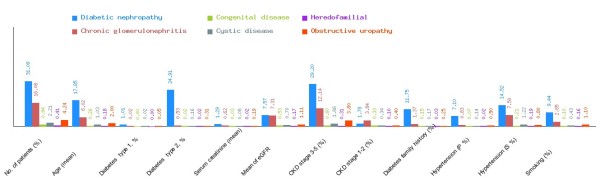
**Overview of various diseases in CDKD.** Graphical representation of diabetic nephropathy, chronic glomerulonephritis, congenital disease, cystic disease, obstructive uropathy and heredofamilial with respect to various parameters.

**Figure 5:**
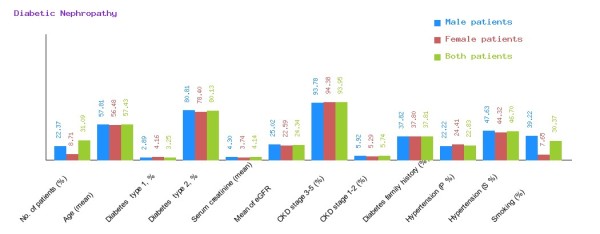
**Graphical representation of diabetic nephropathy in CDKD.** The figure shows a graphical overview of male and female patients of diabetic nephropathy present in the database.

**Figure 6:**
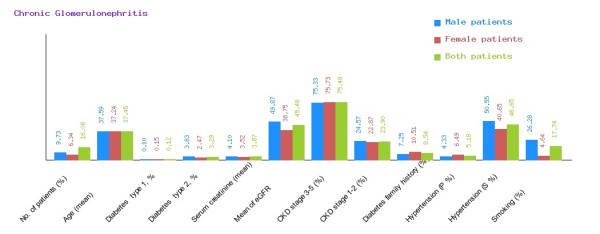
**Graphical representations of chronic glomerulonephritis in CDKD.** The figure shows a graphical overview of male and female patients of chronic glomerulonephritis present in the database.

**Figure 7:**
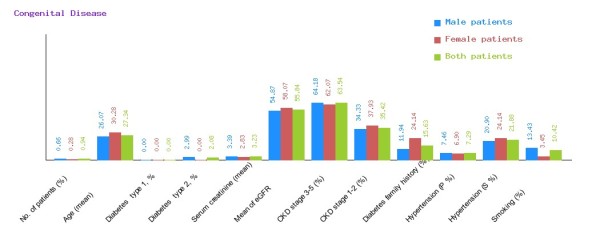
**Graphical representation of congenital disease in CDKD.** The figure shows a graphical overview of male and female patients of congenital disease present in the database.

**Figure 8:**
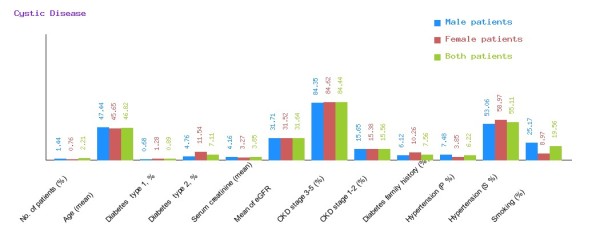
**Graphical representation of cystic disease in CDKD.** The figure shows a graphical overview of male and female patients of cystic disease present in the database.

**Figure 9:**
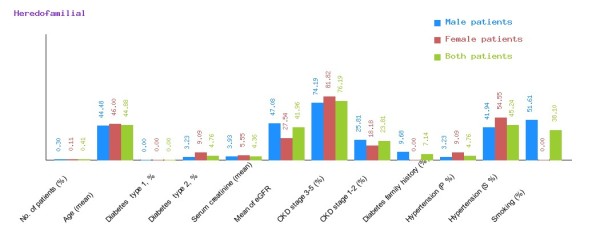
**Graphical representation of heredofamilial in CDKD.** The figure shows a graphical overview of male and female patients of heredofamilial present in the database.

**Figure 10:**
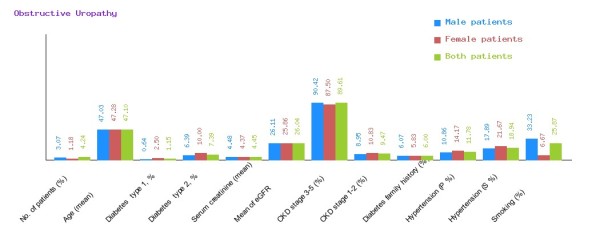
**Graphical representation of obstructive uropathy in CDKD.** The figure shows a graphical overview of male and female patients of obstructive uropathy present in the database.

It is estimated that each year in United States more than 100,000 individuals are diagnosed with kidney disease, a condition in which the kidneys fail to remove the body wastes [2]. Similarly, about 175,000 new patients every year in India develop potentially fatal end-stage renal failure (ESRF) [3]. This generates a huge amount of patient data and requires a proper and efficient way of handling patient record.

India has the largest number of diabetic patients in the world, estimated to be ~ 40.9 million in the year 2007 and expected to increase to ~ 69.9 million by the year 2025 [4]. Nearly 30% of ESRF in India is due to diabetic nephropathy. Diabetic nephropathy is clinically defined by the presence of persistent proteinuria of > 500 mg/day in a diabetic patient [5]. A glomerular filtration rate (GFR) <60 mL/min/1.73 m^2^ for 3 months or more is defined as kidney damage [6].

The first Asian Forum of Chronic Kidney Disease Initiative (AFCKDI) meeting was held in Hamamatsu, Japan in May 2007. Nephrologists from 16 countries of the Asia–Pacific active in the field of chronic kidney disease (CKD) presented 56 papers. Mission of the Asian Forum of Chronic Kidney Disease Initiative was to develop a consensus on a protocol for CKD detection in our region, to analyze risk factors and cost-effective evaluations of proposed interventions, to establish a CKD Initiative network in our region and to contribute to the broader global initiative by using local resources [7].

The exact prevalence of chronic kidney disease in India is not clear in the absence of regular national registry data. The available data is provided only by small observational series or relies on reports from personal experience, but the quality of data is quite uneven [8].

Health-care journals and magazines have touted Electronic Medical records as a means to dramatically improve physician efficiency (productivity and cost) and effectiveness (quality of care). The implementation of an EMR would reduce the need for redundant data collection and allow care providers to quickly review the history and update the medical records wherever necessary. In those cases where the patient or family is unable to provide medical history, having prior data would be invaluable [9].

The fundamental approach of database must be better patient care. The main characteristics of medical records are unrestricted nature, dataflow around the physician/researcher, and statistical analysis of records [10].

The system requires a robust security infrastructure to support authentication, confidentiality, and data integrity. To access the patient record, the patient must be identified uniquely and securely while allowing the physician to visualize, edit and annotate a patient record [11].

Error reduction, quality improvement and lowering of cost can all be achieved through electronic patient record system [12].

In the last decade, costs of computing power hardware have come down, whereas the costs of proprietary software licenses and maintenance fees have remained almost unchanged or increased, limiting the use of such software systems outside developed nations [13]. A potential solution to the barriers of standardisation, cost and business failure is free and open source software [14].

There are many barriers to the acceptance of electronic medical records by physicians. These main categories are: 1. Financial, 2. Technical, 3. Time, 4. Psychological, 5. Social, 6. Legal, 7. Organizational, and 8. Change Process [15].

Physicians who had adopted an EHR consistently reported more positive views of the potential effect of computers on health care than physicians who did not yet have an EHR [16].

## Construction and content

### Database architecture

CDKD was designed as client–server architecture with PHP5 and MySQL, running on an Apache server. PHP, MySQL and Apache technology were preferred as they are open-source components and platform independent. CDKD is hosted on both windows and Linux platform. CDKD eliminates the difficulties of maintaining a DOS based network application on computing facilities at individual hospitals. As a Web based application it is much easier to maintain and support as compared to paper based records. It can be accessible from anywhere and anytime. All graphs are dynamically generated using PHP with the GD library of image functions Figure 1.

### Data collection and organization

The patient record was provided by Department of Nephrology, PGIMER, Chandigarh in excel sheets. In year 2006, the total number of patients was about 14,024. Records where information regarding any parameter (e.g. Serum creatinine) was missing were removed from the dataset.

Finally, we have excluded the data of 3,823 patients with any missing information and analyzed 10201 patient records only.

Data was grouped into six major diseases:

1. Diabetic nephropathy,

2. Chronic glomerulonephritis,

3. Congenital disease,

4. Cystic disease,

5. Obstructive uropathy and

6. Heredofamilial.

Additionally, all records were classified into male and female groups and further subdivided with respect to their age.

Data is broadly divided into two parts i.e. personal details and lab details. A database system is designed to store the personal and laboratory investigatory details of kidney patients. Each patient’s record is divided into three parts (see Figure 2) i.e. personal details, case details and records history. Records history includes patient’s follow-up by physician.

## Utility and discussion

### Database interfaces

The interface of CDKD is designed in a manner which helps user in easy navigation and allows use of various tools integrated in the database. The database interface includes four parts: general user, registered users, administrative users and a super user.

A'. **Researcher interface**: The CDKD database along with its various features is described on the home page, which has links to further navigate the database. All users can access the database through researcher interface and for that there is no need of registration. User can search patient record using case id from search box available in header section. Under the advanced search option, there are four keywords, viz. basic diagnosis, gender, age group and case registration year. User can search all patient records by using any combination of these keywords or by means of at least one keyword. In basic diagnosis textbox user can use full name or part of disease name. Database statistics and tools to calculate eGFR and BMI are available in this section. User network IP address is available on this section.

Estimated glomerular filtration rate (eGFR) is measured by using the new 4-variable Modification of Diet in Renal Disease (MDRD) equation as follows [17]

(1) $$\text{eGFR }=\text{ 186 }\times {\left(\text{serum Cr }\left[\text{mg}/\text{dL}\right]\right)}^{-1.154}\times {\text{ age }}^{-0.203}\times \left(0.742\text{ if female}\right)\text{.}$$

Estimating GFR Using the CKD Epidemiology Collaboration (CKD-EPI) Creatinine Equation as follows [18]

(2) $$\text{eGFR }=\text{ 141 }\times \text{ min}{\left(\text{Scr}/\text{k},1\right)}^{\alpha }\times \text{ max}{\left(\text{Scr}/\text{k},\;1\right)}^{-1.209}\times 0.993^{\text{age}}\times \left(1.018\text{ if female}\right)$$

Where Scr is serum creatinine(mg/dL), k is 0.7 for female and 0.9 for male, Î± is –0.329 for female and –0.411 for male.

Body mass index (BMI) is a measure of body fat based on height and weight. The formulae universally used in medicine produce a unit of measure of kg/m2.

(3) $$\text{BMI }=\text{ mass }\left(\text{Kg}\right)/\left(\text{height}\left(\text{m}\right)\right)\;2$$

B. **Physician interface**: Member’s home page of CDKD consists of links through which physician can annotate patient’s records. This section is restricted to registered users. User can add patient’s personal and laboratory details in patient database and can also edit/update any patient’s record through case id.

Currently, eGFR and BMI calculators are available in this section and a tool for data analysis by which a user can perform analysis of one or more parameters. There are three necessary parameters viz: gender, age group and whether to view all records or the user’s own records.

User can search patient record using case id or basic diagnosis key words from search box available in header section. Physicians can search their own patient’s record through this search box. Identification id provided by them, and can be used only by those who initially provide it.

Under the advanced search option, there are five keywords, viz. all record or my record, basic diagnosis, gender, age group and case registration year. User can search all patient records by using any combination of these keywords or by means of at least one keyword. In basic diagnosis textbox user can use full name or part of disease name.

C. **Administrative user interface:** This area is only accessible to Administrators. From this section administrator can view all registered users. Administrator can edit/update user record. Users Request Box contains each users request for registration and when a user submits a registration form, it will appear here and later it is transferred to user request box.

D. **Super user interface:** This interface is accessible to super user. Super user can make administrators, and can monitor all the steps viz. registration system, which user is registered by which administrator, etc. and at the time of users request for registration an automatic system generated e-mail is sent to super user.

## Description

CDKD currently contains 10,201 data entries i.e. patient records with several kidney disorders like diabetic nephropathy, chronic glomerulonephritis, etc. and is expected to grow substantially as more and more collaboration from within and outside the state are in progress.

A database system is designed to store the personal and laboratory investigatory details of Indian kidney patients. A person’s medical information would be immediately available for the attending physician. Therefore if an individual needs medical care in another part of the country, the attending physicians would have the patient’s entire medical history at their fingertips. Therefore, this system could provide a life saving information that would be invaluable to the attending physicians. The creation of a patient’s database would also allow researchers to track certain diseases as well as to patients’ responses to certain drugs. The emphasis is on experimental results relevant to quantitative renal physiology, with a particular focus on data relevant for evaluation of parameters in statistical models of renal function.

CDKD has been developed with patient confidentiality and data security as a top priority. It can make comparative analysis of one or more parameters of patient’s record and includes the information of about whole range of data including demographics, medical history, laboratory test results, vital signs, personal statistics like age and weight.

This contains highly secure registration system (Figure 3). When a user registers, an e-mail is automatically sent to the super administrator. Only administrators (clinician/physicians) have rights to confirm the user request. After registration, user will receive a confirmation e-mail through administrator and only after this confirmation a user can access the database. This registration is designed in such a manner so that unauthorized users cannot be registered.

There is a security system that supports authentication and secure transmission of personal and confidential patient information. To access the patient record, the patient is identified uniquely and securely through Case ID and Identification Number; Case ID is automatically generated at the time of case entry and Identification Number is provided by physician. As a Web based application it permits physician to see, edit and annotate a patient record from anywhere and anytime. Each physician can see the case details of all patients but not the personal data available in this database.

There is an IP address monitoring system which will track user visit and display current 10 visits on his home page so that a user can monitor his visit.

There are many barriers to the acceptance of electronic medical records by physicians. These main categories are financial, technical, time, psychological, social, legal, organizational, and change process. CDKD is designed as open-source system so there is no financial issue. It eliminates the difficulties of maintaining a DOS based network application and as a web based system all technical issues are solved quickly from our remote server. It is user friendly and only basic level of computer skill is required. The introduction of EMR doesn’t affect the physician’s workflow because system is designed in such a manner so that case registration will take a very short time and other details can be added at any time. EMR can successfully improve patient care or clinical outcomes.

## Statistical analysis

In this work, we have analyzed the records of 10,201 patients (males = 6, 776, and females = 3,425) available in CDKD. Mean age for all patients was observed to be 48.86 ± 16.56 years, 49.69% were male and 47.20% female. In CDKD record, 66.42% individuals were male where as 33.58% were females. Maximum numbers of patients (35.61%) were in age group of 45 to 59 (see Table 1).

Overall, hypertension was present in 7114 subjects (69.74%); 4731 males (46.38%) and 2383 females (23.36%). In 46.38% of male patients, hypertension was primary in 22% and secondary in 39.85% whereas in 23.36% of female patients, primary and secondary hypertensions were seen in 21.63% and 36.99% respectively. Overall, the percentage of secondary hypertension (38.89%) was more than primary hypertension (21.88%). A total of 38.72% of the subjects had diabetes. 41.88% were male and 32.47% were female. Smoking habit was found in 2567 out of 10201 patients; 2294 were male (33.85%) and 273 female (7.97%) (see Table 1).

Mean eGFR was 31.90 in male and 28.08 in female. CKD status was classified into 5 stages on the basis of the eGFR values. A total of 89.03% male patients were classified in the CKD stage 3–5 and 10.97% in the CKD stage 1–2. Percentage of various kidney diseases in 10,102 patients within CDKD is as follows: diabetic nephropathy (30.85%), chronic glomerulonephritis (16.08%), congenital disease (0.94%), cystic disease (2.21%), obstructive uropathy (4.24%) and heredofamilial (0.41%) (see Table 2).

We have categorized CDKD Figure 4 into diabetic nephropathy Figure 5, chronic glomerulonephritis Figure 6, congenital disease Figure 7, cystic disease Figure 8, obstructive uropathy Figure 9 and heredofamilial for Figure 10 statistical analysis relevant to number of patients (%), age (mean ± SE), diabetes type 1, %, diabetes type 2, %, serum creatinine (mean ± SD), mean of eGFR, CKD stage 3–5 (%), CKD stage 1–2 (%), diabetes family history (%), hypertension (primary %, secondary %) and smoking (%) (see Table 2). All statistical tables and graphs are generated dynamically in CDKD. All diseases are further organized into age group of male and female patients.

## Conclusions

The goal of this database is to make kidney-related physiological data easily available to the scientific community and to maintain & retain patient’s electronic record. The purpose of CDKD can be understood as a comprehensive documentation of patient encounters that allows the computerization and reformation of the workflow in health care settings and enhances safety through evidence-based assessment support, quality management, and outcomes treatment. Electronic records may actually be more secure than paper records. Additionally, data from such an electronic database system can be used incognito for statistical reporting in matters such as quality improvement, resource management and public health communicable disease observation.

## Availability of database

The database is publicly available. Any user can access it after a free registration process. The database requires registration to check unauthorized users. CDKD is freely available at http://www.cdkd.org.

## Ethics

This study was approved by the institute ethics committee of the Post Graduate Institute of Medical Education and Research (PGIMER), Chandigarh, INDIA and research was carried out in compliance with the Helsinki declaration.

## Consent

Since no patient identifier appears in the database, the requirement for written consent was waived by the institute’s ethics committee.

## Data protection

Yes, the database adheres to all data protection requirements.

## Abbreviations

BMI: Body mass index; CDKD: A clinical database of kidney diseases; CKD: Chronic kidney disease; eGFR: Estimated glomerular filtration rate; EHR: Electronic health record; EMR: Electronic medical record.

## Competing interests

The authors declare that they have no competing interests.

## Authors’ contributions

SKS developed the database architecture and carried out statistical analysis. AF & SKS created the web interface and performed the validations. The patient records were provided by VJ. AM & VJ conceived the original idea and supervised the work. SKS and AM drafted the manuscript, with SKS in the lead and each author advised on it. All authors read and approved the final manuscript.

## Pre-publication history

The pre-publication history for this paper can be accessed here:

http://www.biomedcentral.com/1471-2369/13/23/prepub
